# Altered mapping of sound frequency to cochlear place in ears with endolymphatic hydrops provide insight into the pitch anomaly of diplacusis

**DOI:** 10.1038/s41598-021-89902-0

**Published:** 2021-05-17

**Authors:** J. J. Guinan, S. M. Lefler, C. A. Buchman, S. S. Goodman, J. T. Lichtenhan

**Affiliations:** 1 Eaton-Peabody Laboratories, Massachusetts Eye and Ear, Boston, MA USA; 2 Department of Otolaryngology, Harvard Medical School, Boston, MA USA; 3 Department of Otolaryngology, School of Medicine, Washington University St. Louis, Campus Box 8115, 660 South Euclid Avenue, Saint Louis, MO 63110 USA; 4 Department of Communication Sciences and Disorders, University of Iowa, Iowa City, IA USA

**Keywords:** Cochlea, Neurological disorders

## Abstract

A fundamental property of mammalian hearing is the conversion of sound pressure into a frequency-specific place of maximum vibration along the cochlear length, thereby creating a tonotopic map. The tonotopic map makes possible systematic frequency tuning across auditory-nerve fibers, which enables the brain to use pitch to separate sounds from different environmental sources and process the speech and music that connects us to people and the world. Sometimes a tone has a different pitch in the left and right ears, a perceptual anomaly known as diplacusis. Diplacusis has been attributed to a change in the cochlear frequency-place map, but the hypothesized abnormal cochlear map has never been demonstrated. Here we assess cochlear frequency-place maps in guinea-pig ears with experimentally-induced endolymphatic hydrops, a hallmark of Ménière’s disease. Our findings are consistent with the hypothesis that diplacusis is due to an altered cochlear map. Map changes can lead to altered pitch, but the size of the pitch change is also affected by neural synchrony. Our data show that the cochlear frequency-place map is not fixed but can be altered by endolymphatic hydrops. Map changes should be considered in assessing hearing pathologies and treatments.

## Introduction

The mammalian cochlea has evolved to have coiled anatomical structures and material properties that gradually vary along the cochlear length so as to produce mechanical filtering that maps sound frequency to cochlear place. In the resulting tonotopic frequency-place map, the largest mechanical responses occur in the cochlear base for high-frequency sounds and in the cochlear apex for low-frequency sounds. The place along the cochlea that is excited by a sound, along with neural temporal synchrony, are the main cues used to translate the physical sound frequency into perceptual pitch. If the maps in the right and left ears are not identical (e.g. from development or subsequent pathology), the result may be right-left pitch differences, or diplacusis.

Diplacusis is a symptom often present in patients with Ménière’s disease, a common inner-ear disorder in adults with a prevalence of 20–40 in every 100,000 people^1^. The primary symptoms of Ménière’s disease include vertigo, tinnitus, aural fullness, and low-frequency sensorineural hearing loss. Diplacusis is present in at least 50% of people with Ménière’s disease, but this prevalence may be an under-estimate because clinicians do not routinely test for diplacusis^2^. A hallmark of Ménière’s disease is endolymphatic hydrops, which is an enlargement of the fluid-filled scala-media duct of the inner ear^3^. In ears with endolymphatic hydrops, excess fluid in the scala media may push on, and thereby stiffen, the basilar membrane (BM), which might change the cochlear frequency-place map and produce diplacusis^4–6^. There are, however, no studies that have shown altered cochlear frequency-place maps in Ménière’s disease.

We created an animal model of endolymphatic hydrops by ablating the endolymphatic sac^7^. At 30 days after the operation, operated ears had normal hearing thresholds at high frequencies and a hearing loss at low frequencies^8,9^, a pattern found in early-stage Ménière’s disease^3^. By using a cochlear perfusion technique that enables assessment of tonotopicity^10,11^, we show, for the first time, abnormal cochlear frequency-place maps in ears that developed normally but have been changed by a disease process (endolymphatic hydrops).

## Results

**Figure 1:**
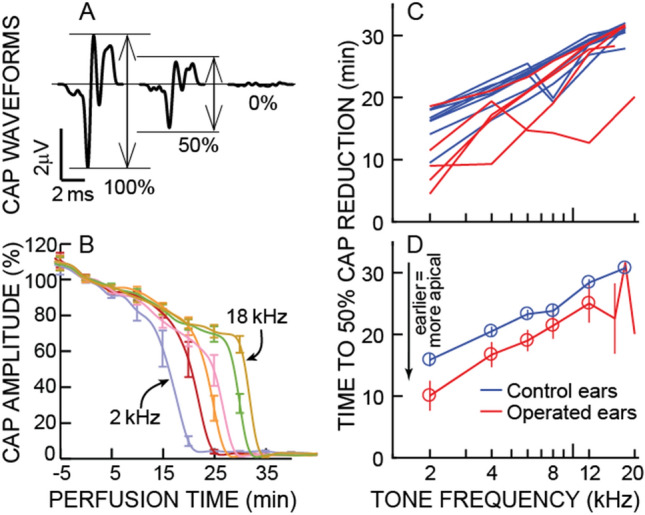
The effects of perfusing kainic acid in artificial perilymph from the low-frequency cochlear apex to the high-frequency cochlear base. (**A**) Example auditory-nerve compound-action-potential (CAP) waveforms at the start of a perfusion (100%), when CAP reduced to 50%, and at the end of the perfusion (0%). (**B**) CAP amplitudes (means from the 8 un-operated control ears, normalized by the average value − 5 to 5 min re perfusion start) evoked by low-level tone bursts (10 dB sensation level (SL) with SL determined by the CAP threshold at each frequency) as functions of time after the start of the perfusion. Error bars represent 1 standard error of the mean (SEM). Color-coded lines show tone-burst frequencies of 2, 4, 6, 8, 12 and 18 kHz. These were reduced in low-to-high sequence (left to right in the figure). (**C**) The perfusion times that produced 50% CAP amplitude reductions as functions of tone frequency. Each line is from one ear: red = operated ears, blue = control. (**D**) Lines show the averages of the data from panel C. Circles indicate the averages are from all ears of a group. Error bars represent 1 SEM (needs at least 2 values). A reduction that is 5 min earlier than normal corresponds to a ~ 1 octave more apical CF location in a normal cochlear map.

In five animals that underwent surgical ablation of the right endolymphatic sac to create endolymphatic hydrops^7^, at 30 postoperative days a solution containing kainic acid was perfused through the cochlea, from apex to base, so that auditory-nerve compound-action-potentials (CAPs) that originated from apical, lower characteristic-frequency (CF) regions, were reduced before CAPs that originated from more-basal, higher-CF regions (Fig. 1A,B). Tone-pip-evoked auditory-nerve CAPs provide frequency-specific objective measures of cochlear neural sensitivity^10^. In eight control (un-operated) animals, the perfusion times at which the CAP amplitudes from low-level tone bursts were reduced to 50% of their original values showed when the perfusion front reached the CF region of the tone^10^. We used animals of either sex weighing ≥ 400 g. In the five sac-surgery animals, the 50% reduction times were *earlier*, on average, than the 50% reduction times of the eight control animals (Fig. 1C,D). For tones 10 dB above the CAP threshold, the control-ear minus operated-ear time differences at 2, 4, 6, 8, and 12 kHz were 5.8, 3.8, 4.3, 2.4 and 3.4 min; the differences were statistically significant for the data from the 5 frequencies taken together (*p* = 0.0157) and at the lowest three frequencies (*p* = 0.019, 0.032, 0.016, 0.145, 0.134, respectively), by a shuffled permutation method (see Methods). The earlier 50% reduction times of the CAPs in the operated animals means that for each frequency, *the mid-point along the cochlea of the origin of the CAPs was at a more apical location* in operated animals than in control animals. Since in normal animals the 50% reduction point is at the CF location, the data indicate that in operated animals the CF location for each frequency was more apical than normal (i.e. the cochlear frequency-place map was shifted from normal).

**Figure 2:**
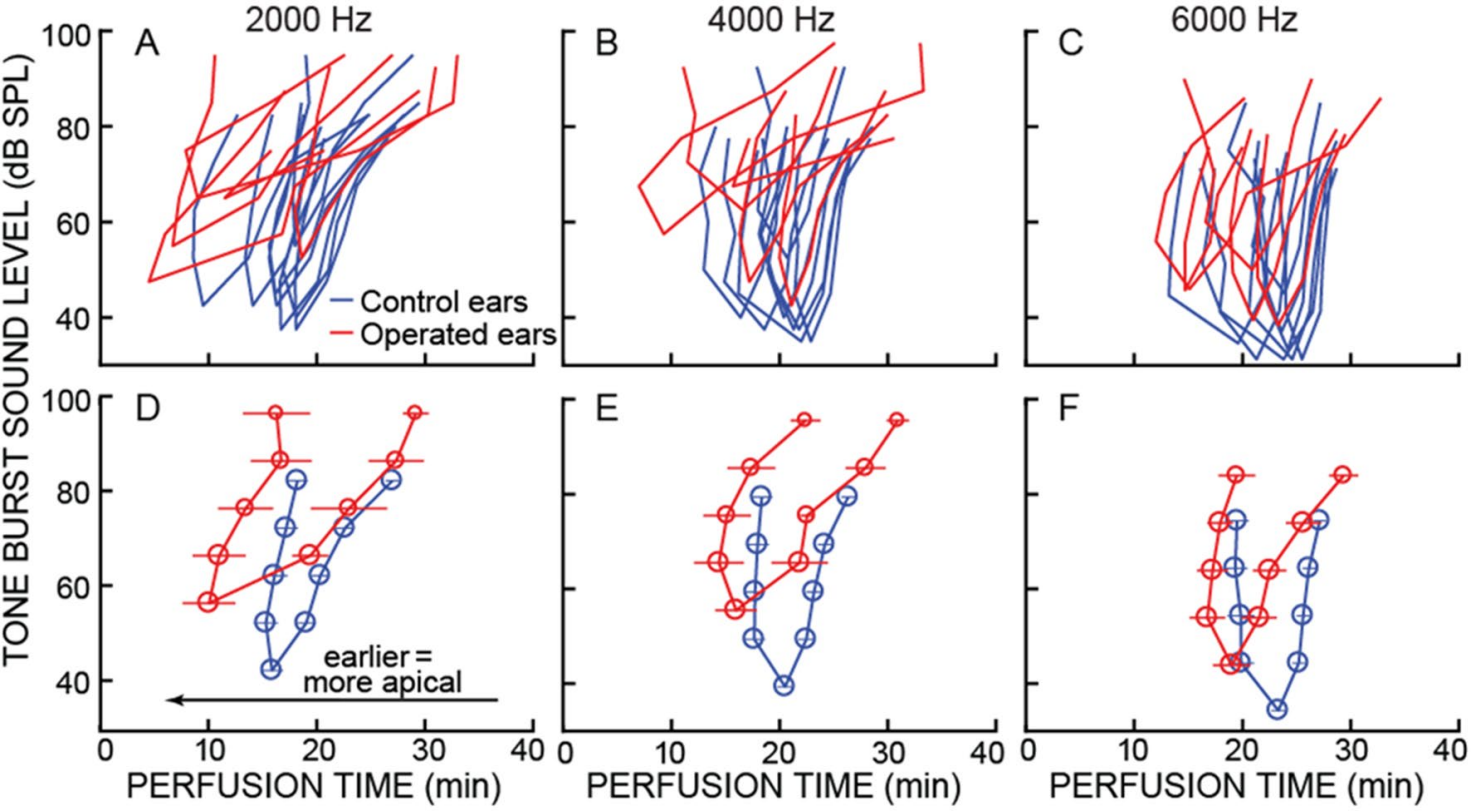
CAP response-reduction-time tuning curves (TCs). Shown are data from the three tone-burst frequencies that, in Fig. 1C,D, had statistically-significant differences between operated and control ears at the 50% reduction points of the 10 dB SL CAPs (the lowest y-axis value of each TC). TC edges are from tone-burst levels that were increased in 10 dB steps above the 10 dB SL point, and show the perfusion times that reduced CAP amplitudes to 80% (left, earlier edge) and 20% (right, later edge) of their pre-perfusion amplitudes. (**A–C**) Each line is the TC from one ear: red = operated ears, blue = control ears. (**D–F**) Lines show the averages of the data from panels A-C. For each group, averages were done across all of the reduction times at a given SL. Large circles indicate the averages are from all ears of a group; medium circles are averages from 4 ears, small circles are averages from 3 ears. Error bars represent 1 SEM.

Abnormalities in cochlear frequency maps can also be appreciated from CAP responses to higher-level tones. CAP response-reduction-time tuning curves (TCs) were calculated from the perfusion times that reduced CAP amplitudes by 20%, 50% and 80%, which show the TC lower-edge, center, and upper edge, respectively (Fig. 2). Four of the five operated ears had CAP response-reduction-time tuning curves with earlier reduction times compared to controls, i.e. their origin along the cochlear length was more apical than normal and in regions where control ears had lower CFs (Figs. 1C, 2). In most operated ears, both low- and high-frequency sides of the TCs were earlier in time (were at more apical locations), but the low-frequency side typically shifted more from control ears than the high side.

**Figure 3:**
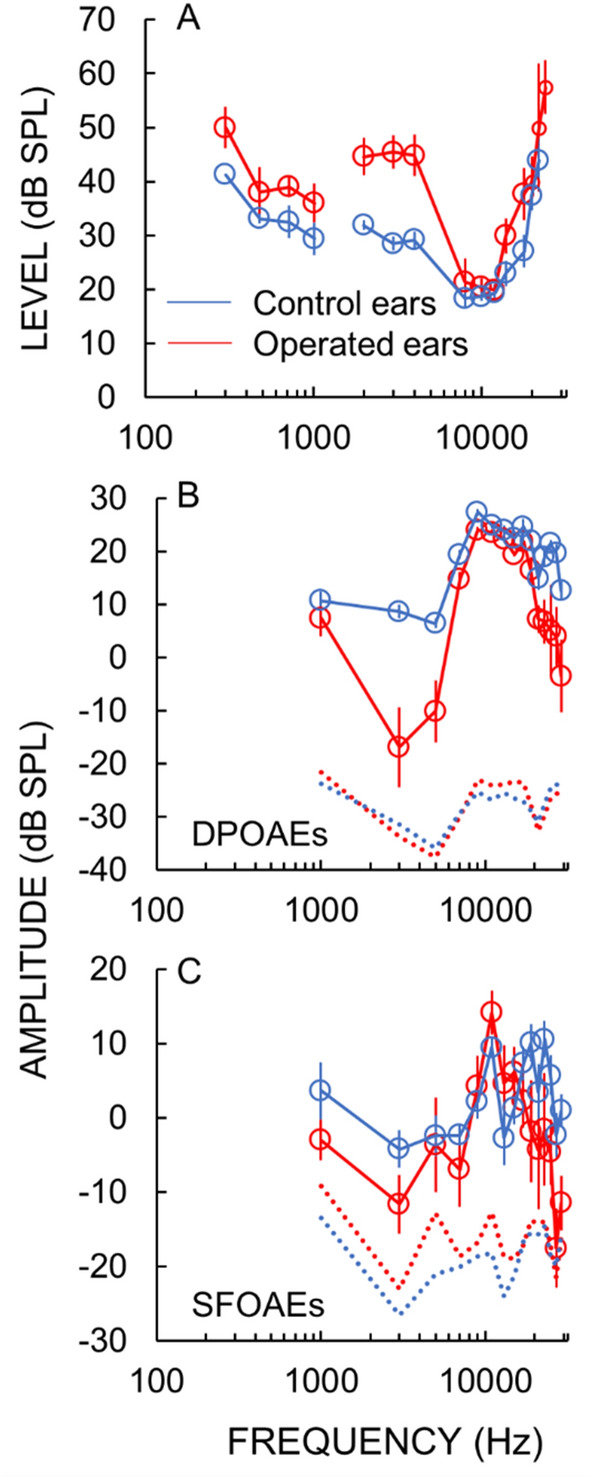
Measurements of hearing function made from control ears (blue) and operated ears (red) before the apical perfusion. (**A**) Hearing threshold measurements were made with the Auditory Nerve Overlapped Waveform (ANOW) at low frequencies (≤ 1 kHz) and auditory nerve compound action potentials (CAPs) at frequencies ≥ 2 kHz. (**B**) Distortion product otoacoustic emission (DPOAE) and (**C**) stimulus frequency otoacoustic emission (SFOAE) amplitude measurements were made before the perfusion. Average DPOAE and SFOAE noise floor measurements are shown as dotted lines.

Operated animals had elevated hearing thresholds at low- (≤ 1 kHz) and mid-(2–4 kHz) frequencies, but thresholds were within normal limits at high-(≥ 8 kHz) frequencies (Fig. 2A). This is an audiometric configuration similar to typical patients early in the progression of Ménière’s disease. In operated animals, otoacoustic emission (OAE) amplitudes were reduced at mid-frequencies (the lowest frequencies tested) but were within normal limits at high frequencies with bigger reductions from normal in distortion-product-OAE (DPOAE) amplitudes than stimulus-frequency-OAE (SFOAE) amplitudes (Fig. 3B,C). OAEs provide noninvasive, frequency-specific objective measures of cochlear mechanical sensitivity^11,12^. These hearing-threshold and OAE results are similar to those we reported previously from other guinea pigs with endolymphatic hydrops^8,9^. It is interesting that similar OAE results were obtained from a patient with Ménière’s disease^12^.

## Discussion

Our results show grossly abnormal cochlear frequency-place maps in animals with experimentally-induced endolymphatic hydrops. In animals with endolymphatic hydrops, the region of the cochlea that responded to mid-frequency tones was sometimes 1–2 octaves more *apical* than usual, i.e. shifted toward regions that normally respond to lower frequencies (Fig. 1C,D). The results support the long-suggested speculation that the diplacusis in Ménière’s-disease patients is due to a change in the cochlear frequency-place map^4–6^. The present report is the first experimental demonstration of an abnormal cochlear frequency-place map produced by endolymphatic hydrops such as occurs in Ménière’s disease. However, because there were a relatively small number of animals in our groups (5 and 8), our data do not provide reliable estimates of the percentage of animals that show map changes or the average shift in the CF-location at each frequency. The results should be viewed an existence proof that operated ears can have changes in their cochlear frequency-place maps.

In neurotypical humans, right-left pitch differences are usually less than one semitone^13–15^. Since the overall frequency range of humans is ~ 10 octaves, a difference < 1 semitone means that the right-left pitch concordance is better than 1% of the frequency range. This does not mean that the concordance in right and left cochlear frequency-place maps is better than 1%. Frequency-place maps originate in the cochlea but the translation of a cochlear tone response to the perception of pitch takes place in the central auditory system and uses cues from both cochlear place and auditory-nerve-fiber periodicity^16^. Whether, or not, the map is different in right and left ears, the tone-induced auditory-nerve periodicity is the same in both ears, so the periodicity cue can be expected to reduce pitch differences arising from any right-left misalignment in cochlear maps (see Supplementary Material section on Spatiotemporal Theories of Pitch). Central plasticity may also help to align inputs from the two ears and thereby compensate for right-left misalignment in the maps during early development. Auditory plasticity also exists in adults, as shown by the changes over many months experienced by cochlear implant recipients^17^. Over time, central plasticity can be expected to reduce the diplacusis that results when an ear is subjected to map changes due to any pathology, e.g. from Ménière’s disease.

Diplacusis is present in people with sensorineural hearing loss, but is seldom more than 1.5 semitones, and tone pitch is most often at a *higher frequency* in the ear with the worst hearing^14,15,18,19^. This is consistent with the hypothesis that the higher sound levels that need to be used in the poorer-hearing ear cause a shift in cochlear place cues toward the higher-frequency cochlear base^6,20^. A basal shift in cochlear place cues as sound level is increased has been shown to be an inherent part of basilar-membrane motion, at least in high-CF regions^21^.

In our experimental animals only one ear was operated on and the other ear was not, so even though we did not test them behaviorally for differences in pitch between the left and right ears, we would expect these animals to have diplacusis. Both the experimental animals and patients with early-stage Ménière’s disease have normal hearing function at high frequencies and a hearing loss at mid to low frequencies. From the correspondence of characteristics in the operated animals and humans with Ménière’s disease, we argue that the operated animals with abnormal cochlear maps would probably have had diplacusis similar to patients with Ménière’s disease. Further, it is reasonable to think that in humans with Ménière’s disease, there are abnormal cochlear maps similar to the maps in operated guinea-pig ears, and that interaural disparity in frequency-place maps is what produces diplacusis.

The operated-ear frequency-place maps shown in Figs. 1C,D and 2 were typically shifted from normal by more than the diplacusis pitch differences reported in Ménière’s disease. In the operated ears, the lowest-level points at the lowest three frequencies had shifts of ½ to 2 octaves of cochlear CF place (Figs. 1C,D, 2). In contrast, Ménière’s-disease diplacusis is usually ¼ octave or less, with a few cases in the range of ½ octave or more^5,18,19^. There are several possible reasons why the map shifts in our operated-ears are larger than the typical Ménière’s diplacusis: (1) Ménière’s disease is bilateral in about 1/3 of patients, which would reduce right/left pitch differences. (2) Our measurements were in ears soon after the induction of endolymphatic hydrops, while patients often do not present to the clinic until the problem has become substantial which may allow time for central adaptation. (3) Our guinea-pig pathology may differ from human Ménière’s disease, which probably has multiple origins^22^. (4) Auditory-nerve periodicity may offset right-left cochlear-map differences in determining pitch.

Most patients with diplacusis and unilateral Ménière’s disease (77%) perceive pitch to be lower in the affected ear^14,18,19^, which is opposite the direction found in ears with typical high-frequency sensorineural hearing loss (see above). Edolymphatic hydrops in Ménière’s ears has been hypothesized to be associated with an increased scala-media pressure that pushes on the organ of Corti and stiffens the BM^4,6,19,23^. In cochlear models, an increase in BM stiffness increases the local the resonant frequency^21^, which would shift CFs apically, a direction consistent with our results and the usual diplacusis in Ménière’s disease.

Our results have implications for the vestibular system, as patents with endolymphatic hydrops often have both auditory and vestibular symptoms. Vestibular sensitivity to motion depends on an end organ’s ratio of mass to stiffness, which sets the resonant frequency. Increased endolymph can be expected to push on vestibular end organs and increase their effective stiffness, which increases the resonant frequency of the saccule as shown by vestibular evoked myogenic potentials^24–27^. The vestibular central nervous system (CNS) has a great capacity to adapt to changes in the peripheral end organs, but over a period of days. If an end-organ resonant frequency changes too fast (such as during the fluctuating symptoms of Ménière’s disease that occur over periods of minutes to hours), adaptation of the vestibular CNS may not be able to keep up, resulting in a mismatch between peripheral vestibular signals and CNS assessing of these cues. This mismatch may lead to the vestibular symptoms of Ménière’s disease. Similar mismatches between the auditory periphery and the decoding of auditory cues in the auditory CNS may lead to diplacusis.

Pathologies other than Ménière’s disease may also produce map alterations and diplacusis. Any pathology that results in endolymphatic hydrops (e.g. semicircular canal dehiscence syndrome^28^), or otherwise changes the effective stiffness of the BM or the mass of the organ of Corti (e.g. through inflammation) could potentially change the cochlear frequency-place map. The extent of map shifts and diplacusis in other diseases that affect the inner ear is a little explored area. Diplacusis can be easy to measure and could be a useful tool in the diagnosis of a variety of pathologies and in tracking treatment efficacy.

## Methods

### Animal preparation

Animal preparation methods on have been detailed in our previous publications^7–9,29^, and here we provide essential information. Guinea pigs were sedated with intraperitoneal injection of 100 mg/kg Inactin hydrate (i.e., thiobutabarbital sodium). The fur on the head and neck were shaved, and a tracheotomy was done to allow artificial ventilation and to maintain anesthesia with ~ 1.2% of isoflurane supplemented with oxygen gas, adjusted to maintain 5% end-tidal CO_2_. Pulse oximeter measurements were used to monitor O_2_ saturation, expired CO_2_ level, and heart rate. Body temperature was kept at 38 °C using a heating blanket and rectal thermometer. The head was secured with a bite bar, snout clamp, a hollow ear bar on the right side, and a solid ear bar on the left side. The animal was placed in the supine position, and a cannula was inserted into the jugular vein on the left side. Hydration was maintained by administering lactated Ringer solution (0.5 mL/h) through the cannula. The right bulla was accessed ventrally by removing soft tissue and the jaw. The ear canal was cut on the right side to allow placement of the hollow ear bar with no acoustic leaks. The animal was kept in a double-walled sound-treated chamber that was heated so the area immediately around a guinea pig’s head was ~ 25 °C. All procedures were approved by the Washington University Institutional Animal Care and Use Committee. All methods were carried out in compliance with the ARRIVE guidelines: http://www.nc3rs.org.uk/page.asp?id=1357. We confirm that all methods were carried out in accordance with relevant guidelines and regulations.

### Sound presentation and calibration

Here we provide brief information on sound presentation and calibration, but details can be found in our previous publications^8–10^. A 1/8″ reference microphone (GRAS type 40P) and custom-made coupling system was used to set stimulus levels in the guinea pig ears. The transfer function of the hollow ear bar (5 cm, 0.322 cm i.d.) was used to calibrate sound stimuli. The end of the hollow ear bar that sits closest to the tympanic membrane was coupled to a simulated ear canal that had the appropriate dimensions and volume of a cut guinea pig ear canal. The reference microphone was placed approximately where the tympanic membrane would be. The ER-10X was coupled to the other end of the hollow ear bar. Stimuli were presented through the ER-10X loudspeakers into the hollow ear bar and measured by the reference microphone. The measured transfer function of the hollow ear bar was applied to calculate the pressure at the tympanic membrane for all stimuli used in the experiment.

The ER-10X probe microphone was used for recording otoacoustic emissions (not for setting in situ ear-canal sound levels). The ER-10X microphone was calibrated with a long copper tube (1.83 m; 0.635 cm i.d.) closed at one end and coupled to a sound source at the other end. The probe and reference microphones were sealed into small holes located ~ 2.5 cm from the sound source. These microphones were positioned opposite each other and perpendicular to the long axis of the tube. The microphone inlet (probe) and diaphragm (reference) were positioned to be flush with the wall of the tube. A train of click stimuli was presented through the loudspeaker, and the incident wave was simultaneously measured with both probe and reference microphones. The inter-click interval was spaced to allow internal reflections within the tube to decay into the noise floor before the next click was presented. Measurements were averaged and windowed to include only the incident wave. A flattened probe response from 0.1 to 34 kHz was calculated using the transfer function relating probe and reference microphones.

### Neural measurements

Method details for neural measurements have been described^8,9,11^. Briefly, neural measurements (e.g. Figs. 1A, 3A) were made with Ag/AgCl ball electrode in the round-window niche (non-inverting), and platinum needle electrodes positioned into the musculature of the right jaw (inverting) and neck (ground). Responses were band-pass filtered at 0.1–3 kHz, and amplified 10,000 times (GRAS CP511; AstroNova, West Warwick, RI). Sampling frequency was 96 kHz.

Auditory Nerve Overlapped Waveform (ANOW) measurements were made with tone bursts (33.3 ms duration) alternating in polarity (92 repetitions) at 300, 480, 720, and 1020 Hz^30–32^. The first 5 ms and final 8.3 ms of each ANOW waveform were removed and the remaining 20 ms was ramped on and off, with a ramp duration = 1/*f* sec, where *f* is the tone burst frequency. ANOW waveforms were computed by taking 0.5 times the sum of the responses from the two tone burst phases.

For CAP measurements, tone bursts (1.0 ms rise/fall, 13.9 ms duration) were presented in alternating polarity (128 repetitions) and interleaved with three periods of silence of the same duration, for a total of 69.5 ms and a repetition rate of 14.38 / second. Tone bursts were varied from 80 to 10 dB SPL in 5 dB steps.

The CAP threshold for each frequency was determined as the sound level that evoked a 10 µV peak-to-peak CAP amplitude using the stimuli described in the previous paragraph. During the perfusion, tone bursts at these test frequencies were presented at their threshold level and in 10 dB increments above threshold level. CAP measurements were made in some animals with enough responses averages at each level to achieve a signal-to-noise ratio (SNR) of ~ 6 dB (fewer responses at higher levels), and in other animals with a fixed number of averages. The whole sequence was repeated every ~ 1 min starting 5–10 min before the perfusion and continuing throughout the perfusion.

### Otoacoustic emission (OAE) measurements

Our OAE methods have been detailed^8,11^. Briefly, DPOAEs at 2*f*_1_*–f*_2_ were measured using two tones with one-second durations, a frequency ratio *f*_2_/*f*_1_ = 1.22, and levels *L*_1_, *L*_2_ of 60 and 50 dB SPL, respectively (Fig. 3B). *f*_2_ ranged from 1 to 30 kHz in 2 kHz steps, with 12 stimulus repetitions presented at each frequency. SFOAEs were elicited with 250 ms (10 ms rise/fall), 40 dB SPL tones, using the double-evoked method with suppressor tones of 60 dB SPL that were 50 Hz above the SFOAE frequency^33–35^ (Fig. 3C). Probe tones ranged from 1 to 30 kHz in 2 kHz steps and were presented 24 times at each probe frequency. Noise floors for OAEs were estimated as the standard error of the amplitudes converted to dB SPL.

### Apex to base perfusion methods

Method’s details for the apex-to-base perfusion have been described^29^. During the apex-to-base perfusion, the solution (2.16 mM kainic acid in artificial perilymph) was driven through scala tympani to the cochlear aqueduct in the cochlear base by a programmatically-controlled ultrapump (UMP3; World Precision Instruments) with the pump rate varied under computer control at 1-min intervals to achieve a constant flow of 0.5 mm/min (~ 5 min per CF octave^29^) along the scala tympani (which has a varying cross-sectional area). While endolymphatic hydrops can greatly decrease the cross-sectional area of scala vestibuli, hydrops does not change the cross section of scala tympani and thus does not affect the flow of fluid through scala tympani.

Perfusion with kainic acid blocks neuroexcitation at the inner-hair-cell to auditory-nerve synapse^36–38^, but does not affect outer hair cells and cochlear amplification (as shown by the kainic acid solution having no effect on OAEs^29^. Our constant flow rate of 0.5 mm/min enables us to determine location along the cochlea where CAP measurements originate. From the guinea pig cochlear-frequency place map (CF place vs distance along the cochlea^39^) and the time at which the perfusion reduced the CAP amplitude, we can calculate the location of the perfusion front along the length of scala tympani. Thus, the time at which a measurement was reduced can be translated into the location along the cochlea that gave rise to the response. On average, kainic acid reduced tone-evoked CAP amplitudes by half at approximately the same perfusion time that an independent calculation of the fluid front location reached the tone CF location^10^. Since the CAP response to a low-level tone arises at the CF region, the concurrence of perfusion results with the calculation of the fluid-front-position’s CF demonstrates that the perfusion method is an accurate method for identifying where along the cochlea the response originates.

### Constructing CAP reduction-time tuning curves

Our methods for constructing CAP reduction-time tuning curves have been detailed^10^. The sound level needed to produce a 10 μV CAP amplitude was measured and used to determine “hearing threshold” at each frequency tested (2, 4, 6, 8, and 12 kHz). At each level above the threshold level, the perfusion time required to reduce the response to 80% of the original was taken as showing the apical edge of the region from which the CAP originated, and the time required to reduce the response to 20% of the original was taken as showing the basal edge of the region from which the CAP originated^10,11,30^. The perfusion time required to reduce the threshold level response to 50% was taken as the time at which the solution reached the center of the region from which the CAP originated (which could be thought of as the “tip” of the response region). 20% and 80% reductions at this lowest level could not be determined because the SNRs of the responses were not good enough.

In operated ears, the low- and mid-frequency thresholds were higher than in control ears, but this seems unlikely to have caused the tuning curves of the operated animals to have shifted to more apical positions. In the normal-ear tuning curves, for low frequency tone bursts as sound level was increased, the response area became wider and shifted basally, not apically^10^. For frequencies 2–4 kHz, as sound level was increased, the low-frequency TC edge moved somewhat more apical, the high-frequency TC edge moved much more basal and, the 50% reduction points generally moved basally^10^.

### Endolymphatic hydrops: histology

**Figure 4:**
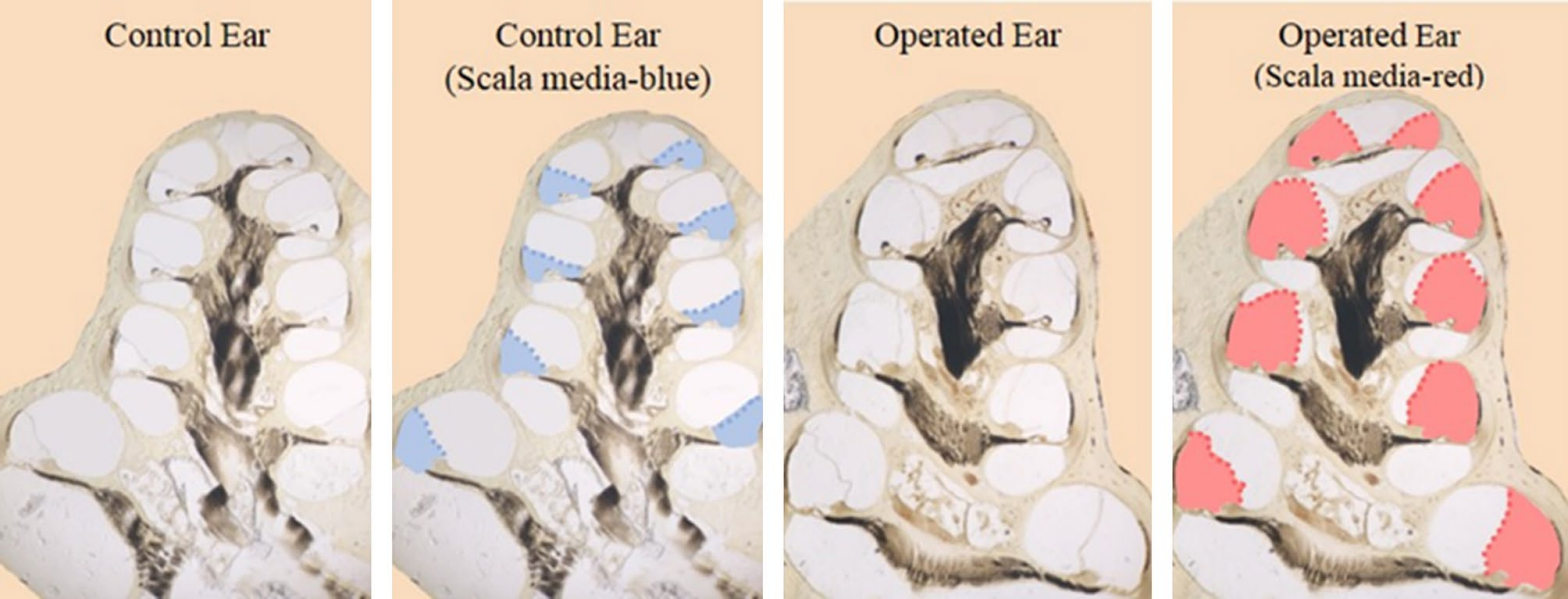
Mid-modiolar histological sections of representative control (left panels) and operated (right panels) cochleae of guinea pigs that underwent a surgery to ablate the right-side endolymphatic sac and create endolymphatic hydrops. Colored shading identifies the scala media of the left (blue) and right (red) ears. Scala media cross-sectional area was enlarged in the right ear, demonstrating endolymphatic hydrops.

Our methods for histological assessment of endolymphatic hydrops have been described^8^. At the end of the cochlear perfusion, sodium pentobarbitol was administered intraperitoneally (250 mg/kg), and temporal bone fixation was achieved by transcardiac perfusion with 0.1 M phosphate buffer (~ 30 mL, pH 7.4) followed by 2% paraformaldehyde and 0.5% glutaraldehyde in 0.1 M phosphate buffer (~ 60 mL, pH 7.4). Temporal bones were stored for at least 12 h in fixative at 4 °C. Cochleae were then decalcified with 0.1 M EDTA in 0.1 M phosphate buffer for 6 days at room temperature. The pH of all solutions was adjusted to 7.4. After decalcification, cochleae were post-fixed (1% OsO4 in dH2O) for 1 h, dehydrated in a graded ethanol series and propylene oxide, and then embedded in Araldite resins. The plastic blocks were sectioned in a vertical plane parallel to the modiolus at 40 µM with a carbide steel knife. Each section was mounted on microscope slides in Permount, coverslipped, and visualized using an OlympusBX51 microscope and DP71 camera (Olympus America Inc.). Scala media cross-sectional area was quantified in each cochlear half turn of a mid-modiolar image using Image J. Examples of a control ear and an ear with endolymphatic hydrops are shown in Fig. 4.

### Statistical tests of operated-ear versus control-ear response-reduction times

To determine if the 50% response-reduction times of the operated ears were significantly different from the 50% response-reduction times of the control ears, we used permutation tests (ANOVA was not used because the data were not normally distributed—although ANOVA gave results not far different from those obtained below). Separate tests were done at each of the five frequencies for which there were data for all 5 operated ears and all 8 control ears. The data from each frequency formed a set and was tested separately. The grand average of the differences at all five frequencies formed an additional set and was tested separately. For each data set, the statistic of interest was {the average of the 50% reduction times of the control ears} minus {the average of the 50% reduction times of the operated ears}. Each original (unshuffled) data set yielded a set difference that will be called the original set difference (OSD).

For each set of data, the 50% reduction times of all of the ears included in the original averages of that group (N = 5 operated ears and M = 8 control ears) were pooled (yielding N + M = 13 ears for the single-frequency data sets). These formed the input ears for the statistical tests. There are 1287 unique ways to choose five elements from 13 elements (1287 = 13!/(8! * 5!), where “!” denotes the factorial value). If in the grand-average multi-frequency test, animal numbers are randomized separately at each frequency, there are 1287 to the 5^th^ power (= 3.5 * 10^15^) unique ways to choose the elements. So that the grand-average and single-frequency statistical tests could be done in the same way (and because it was easier to program), we computed the probabilities using random permutations done multiple times, which for a large number of repetitions converges on the same probability as the exact permutation computation (i.e. using each unique permutation once and only once).

To test the null hypothesis that the data from operated ears and control ears came from identical distributions, the data were combined into a single superset containing N + M data points. New-sets of N pseudo-operated ears and M pseudo-control ears were formed by randomly permuting the list of animal numbers of the N + M ears in the superset and choosing the first N in the permuted list to be pseudo-operated ears and the remaining M ears to be pseudo-control ears. For each permuted list we computed the difference, PD = {pseudo-control-ear average 50% reduction time} minus {pseudo-operated-ear average 50% reduction time}. The grand-average multi-frequency set was analyzed in two ways: (1) at each frequency the animal numbers were separately and independently permutated and then the PDs from the five frequencies were added, and (2) the same permutation of animal numbers was used at all frequencies and the PDs from the five frequencies were added. PD (or the sum of the PDs for the multi-frequency set) was compared to the original set difference, and if PD ≥ OSD, the hypothesis that the operated ear and the control ear data were drawn from the same distribution was scored as false. We did a one-sided test comparison because to account for the direction of the most common diplacusis in Meniere’s disease, the operated ears had to be reduced earlier than the control ears. For each data set, a permutation and PD ≥ OSD test was done 10^7^ times. The number of false trials divided by 10^7^ was the probability that that the OSD value could be obtained with the operated-ear and control-ear data obtained from the same distribution, i.e. a low p value indicates that it is unlikely that they came from the same distribution. In other words, p is the statistical significance that the 50% response reduction times of the operated ears and the control ears are different.

For the grand average of the differences at all five frequencies, *p* = 3.8*10^−5^ for method 1 and *p* = 0.0157 for method 2. Since these are statistically significant at the 0.05 level, it indicates that the overall difference in reduction times was statistically significantly different. We then considered each frequency separately to determine which frequencies might individually be statistically-significantly different. For 2, 4, 6, 8, and 12 kHz, *p* = 0.019, 0.032, 0.016, 0.145, 0.134, respectively (If we had used a two-sided test, the results would be *p* = 0.032, 0.044, 0.018, 0.295, 0.142, respectively). Thus, the single-frequency differences were significant at the 0.05 level for 2, 4 and 6 kHz. It is interesting to note that with method 2, the grand-average probability was only slightly lower than the smallest probability at one frequency. This is because the variation across ears was highly correlated across frequency. A clear example of this can be seen in Figs. 1 and 2 where one operated ear did not show a map shift and was like the control ears *at all frequencies*. This ear had a disproportionate effect on the calculated probabilities and illustrates that the probabilities depend on the particular sample of animals in each group.

### Approval for animal experiments

All procedures were approved by the Washington University Institutional Animal Care and Use Committee. All methods were carried out in compliance with the ARRIVE guidelines: http://www.nc3rs.org.uk/page.asp?id=1357.

## Supplementary Information

Supplementary Information.
