# Supplementary material for: Altered mapping of sound frequency to cochlear place in ears with endolymphatic hydrops provide insight into the pitch anomaly of diplacusis
Source: Sci Rep. 2021 May 17;11:10380. doi: 10.1038/s41598-021-89902-0 (PMC8128888; doi:10.1038/s41598-021-89902-0)
Supplement: Supplementary file 1 — Supplementary Information. [file 41598_2021_89902_MOESM1_ESM.docx]

Supplementary Information

Altered mapping of sound frequency to cochlear place in ears with endolymphatic hydrops provide insight into the pitch anomaly of diplacusis

by

J.J. Guinan Jr ^a^, S.M. Lefler ^b^, C.A. Buchman^b^, S.S. Goodman ^c^ & J.T. Lichtenhan ^b^

^a^ Eaton-Peabody Laboratories
Massachusetts Eye and Ear
Boston, Massachusetts, USA
Harvard Medical School
Department of Otolaryngology
Boston, Massachusetts, USA

^b^ Washington University St. Louis, School of Medicine
Department of Otolaryngology
Saint Louis, Missouri, USA

^c^ University of Iowa
Department of Communication Sciences and Disorders
Iowa City, Iowa, USA

^*^ Corresponding author

Jeffery T. Lichtenhan

Department of Otolaryngology, Campus Box 8115

Washington University St. Louis School of Medicine

660 South Euclid Avenue

Saint Louis, MO 63110 USA

jlichtenhan@gmail.com

## Map change in development versus map change from later pathology

Cochlear frequency-place maps that are different from normal cochlear maps have been produced in genetically modified mice in which the genes for key structural proteins in the cochlea were knocked out^1,2^. These genetic modifications changed the normal development and structure of the cochlea. It is not surprising that ears that have had their structure modified would have modified cochlear frequency-place maps. Such animals would be expected to have similar anatomy in right and left ears and not have diplacusis. Although the genetically modified animals had abnormal cochlear frequency-place maps^1,2^, this kind of map change is fundamentally different from an abnormal map produced by a pathology in a cochlea that had developed normally and that had a normal cochlear frequency-place map at one time.

A recent analysis has argued that cochlear tuning need not be derived from local cochlear micromechanics (as is usually thought) because the basic features of cochlear tonotopic maps can be produced by the gross shape of cochlear spiral anatomy (the scale taper from large in the base to small in the apex, along with BM width tapering in the opposite direction)^3^. However, this analysis does not rule out important contributions from cochlear micromechanics. Indeed, our work shows large changes in the tonotopic map without changes to the gross anatomy of the cochlear spirals.

There are several kinds of changes that are best viewed as disruptions of the cochlear frequency-place map, rather than a changed mapping of sound frequency to cochlear place. Various causes, particularly loud sounds, can cause cochlear pathology (e.g. loss of inner hair cells or connections to auditory-nerve fibers) that makes regions of the cochlea unable to transmit clear signals to the central nervous system (CNS). These cochlear “dead regions” can produce a variety of changes in the place along the cochlea that responds best to a given tone, but the resulting changes in the signals sent to the CNS are best understood as “off-place listening” brought about by the dead regions rather than by changes in the cochlear maps^4^. Another kind of tuning change, most apparent with broad-band sounds, has been called “distorted tonotopy”, DT^5^. This effect appears to be due to decreased tip-to-tail ratios in auditory-nerve tuning curves brought about by acoustic trauma. In response to a broad-band sound, the neuron is excited more by tail-frequency energy than by tip-frequency energy. In DT, tonotopic mapping is disrupted but through a loss of tonotopic specificity, rather than a change in the cochlear tonotopic map.

It has recently been found that in low-CF regions (e.g. <1 kHz) of experimental animals: (1) gross cochlear motion has an approximately low-pass characteristic, (2) low-CF mechanical tuning changes little with sound level, and (3) the band-pass tuning of low-CF auditory-nerve fibers comes from an additional high-pass process after the gross transverse motion in the cochlea^6,7^. This pattern is substantially different from that found at high frequencies^8^ and can be expected to influence pitch perception. Endolymphatic hydrops could change these motions and affect diplacusis, but current knowledge is insufficient to say how.

## Spatiotemporal Theories of Pitch and shortened delays in Ménière’s disease

In the classic analysis of pitch, “cochlear response place” and “neural synchrony” provide cues that are analyzed separately in the central nervous system and are then combined to determine pitch. In spatio-temporal theories of pitch, cochlear-place cues and synchronization cues are detected together in a way that takes into account that the synchronizations of auditory-nerve fibers are spread out in time because of greater traveling wave delays for more apically located, low-CF auditory-nerve fibers^9,10^. In applying spatio-temporal theories of pitch to Ménière’s disease, a possible complication is that the speed of the traveling wave may be different in Ménière’s ears than in normal ears. Studies of the latencies of auditory-brainstem-response wave-V’s and cochlear CAP responses found unusually short latencies in Ménière’s ears^11–14^. However, the interpretation of these results is not straightforward. For a tone, the speed of the overall traveling wave is not a constant along the cochlea, instead, the speed is very fast from the stapes to near where the traveling wave peaks and then it greatly slows^15^. Furthermore, there is evidence that for a single tone there is more than one traveling wave, that these traveling waves interact, and that this interaction may determine where cochlear amplification takes place along the cochlea^7^. An increase in BM stiffness due to endolymphatic hydrops would be expected to change many things: the location of the basilar-membrane response peak, the local speeds of the traveling waves, how the traveling waves interact, where there is cochlear amplification, the spatial origins of otoacoustic emissions along the cochlear length, and perhaps many other aspects of the mechanical response of the cochlea. Changes in any of these mechanical response properties can be expected to affect the pattern of auditory-nerve-fiber responses. Thus, unusually short latencies of physiologic measurements made from Ménière’s diseased ears does not provide definitive information about the origin along the cochlea of the measurements. For instance, a stiffening of the BM caused by the endolymphatic hydrops could speed up the BM traveling wave so that the shorter latencies in Ménière’s diseased ears is compatible with the more apical response origins that we have found. More knowledge about cochlear mechanics is needed before we understand the properties of the multiple cochlear traveling waves, how they lead to excitation of inner hair cells, and the implications of these cochlear-mechanics complexities for theories of pitch and the latencies of physiologic responses.

## Possible future experiments

It seems reasonable to hypothesize that in the perception of pitch and diplacusis, tone-response right-left cochlear-map differences may be counteracted and/or lessened by the auditory-nerve periodicity in response to a tone being the same in both ears. The extent to which diplacusis is influenced by synchrony being the same in right and left ears might be tested by reducing synchrony in one ear from that in the other. Tests could be done on Ménière’s disease patients at a frequency that the patient shows a significant diplacusis for tones. Tests could be done using (1) tones (to establish the baseline diplacusis), (2) uncorrelated narrow-band-noise, (3) correlated narrow-band noise, and (4) narrow-band noise in one ear and a tone in the other. Instead of tones and narrow-band noise, such tests could be done with harmonic stimuli compared to noise stimuli with similar spectra. There are many possible outcomes from comparing the diplacusis with various combinations of these stimuli. To highlight one: If the diplacusis measured with uncorrelated narrow-band-noise in the two ears was greater than that with correlated narrow-band-noise, it would indicate that the synchrony cue has a substantial influence and was being compared across ears in the auditory CNS.

Diplacusis can be relatively easy to measure quantitatively in humans and insight may be gained by determining the correlation of diplacusis with other metrics, e.g. otoacoustic emissions, and/or sound thresholds. According to a recent paper^16^, 27% of ears with superior semicircular canal dehiscence syndrome (SSCDS) have endolymphatic hydrops, which suggests that they might have diplacusis and, if so, that diplacusis could be an ancillary test that helps to reach a diagnosis of SSCDS.

## Supplementary References

1. Russell, I. J. *et al.* Sharpened cochlear tuning in a mouse with a genetically modified tectorial membrane. *Nat. Neurosci.* **10**, 215–223 (2007).

2. Russell, I. J. *et al.* Emilin 2 promotes the mechanical gradient of the cochlear basilar membrane and resolution of frequencies in sound. *Sci. Adv.* **6**, 1–14 (2020).

3. Altoè, A. & Shera, C. A. The cochlear ear horn: geometric origin of tonotopic variations in auditory signal processing. *Sci. Rep.* **10**, 20528 (2020).

4. Moore, B. C. J. Dead Regions in the Cochlea: Conceptual Foundations, Diagnosis, and Clinical Applications. *Ear Hear.* **25**, 98–116 (2004).

5. Parida, S. & Heinz, M. G. Noninvasive Measures of Distorted Tonotopic Speech Coding Following Noise-Induced Hearing Loss. *J. Assoc. Res. Otolaryngol.* **22**, 51–66 (2021).

6. Recio-Spinoso, A. & Oghalai, J. S. Mechanical tuning and amplification within the apex of the guinea pig cochlea. *J. Physiol.* **595**, 4549–4561 (2017).

7. Guinan, J. J. J. The interplay of organ-of-Corti vibrational modes, not tectorial- membrane resonance, sets outer-hair-cell stereocilia phase to produce cochlear amplification. *Hear. Res.* **395**, 108040 (2020).

8. Robles, L. & Ruggero, M. A. Mechanics of the mammalian cochlea. *Physiol. Rev.* **81**, 1305–1352 (2001).

9. Shamma, S. A. Speech processing in the auditory system II: Lateral inhibition and the central processing of speech evoked activity in the auditory nerve. *J. Acoust. Soc. Am.* **78**, 1622–1632 (1985).

10. Oxenham, A. J. How We Hear: The Perception and Neural Coding of Sound. *Annu. Rev. Psychol.* **69**, 27–50 (2018).

11. Horner, K. C. & Cazals, Y. Rapidly fluctuating thresholds at the onset of experimentally-induced hydrops in the guinea pig. *Hear. Res.* **26**, 319–325 (1987).

12. Thornton, A. R. & Farrell, G. Apparent travelling wave velocity changes in cases of endolymphatic hydrops. *Scand. Audiol.* **20**, 13–18 (1991).

13. Donaldson, G. S. & Ruth, R. A. Derived-band auditory brain-stem response estimates of traveling wave velocity in humans: II. Subjects with noise-induced hearing loss and Meniere’s disease. *J. Speech, Lang. Hear. Res.* **39**, 534–545 (1996).

14. Don, M., Kwong, B. & Tanaka, C. A diagnostic test for Ménière’s disease and cochlear hydrops: Impaired high-pass noise masking of auditory brainstem responses. *Otol. Neurotol.* **26**, 711–722 (2005).

15. Patuzzi, R. in *The Cochlea* (ed. Dallos, P.J., Popper, A.N., Fay, R. R.) 186–257 (Springer-Verlag, 1996).

16. Ray, A. *et al.* MRI contribution for the detection of endolymphatic hydrops in patients with superior canal dehiscence syndrome. *Eur. Arch. Oto-Rhino-Laryngology* (2020) doi:10.1007/s00405-020-06282-3.
